# Supplementary material for: Impact of Body Mass Index on the Survival of Patients with Sepsis with Different Modified NUTRIC Scores
Source: Nutrients. 2021 May 30;13(6):1873. doi: 10.3390/nu13061873 (PMC8226650; doi:10.3390/nu13061873)
Supplement: Supplementary file 1 [file nutrients-13-01873-s001.zip › nutrients-1240178-supplementary.pdf]

Supplementary Material

Table S1. Baseline characteristics and outcomes of patients with sepsis in the validation cohort of the study

mNUTRIC score on the first day	All (n=492)	mNUTRIC score ≥6 (n=245)	mNUTRIC score ≤5 (n=247)	*p*	Underweight (n=81)	Normal-weight (n=241)	Overweight (n=170)	*p*
**Demographic characteristics, mean ± SD**								
Age (years)	68.7 ± 14.5	72.8 ± 12.0	64.7 ± 15.6	<0.001***	71.8 ± 14.3	67.7 ± 15.3	68.7 ± 13.3	0.207
BMI, kg/m^2	23.7 ± 6.0	24.1 ± 5.7	23.3 ± 6.3	0.032*	16.0 ± 1.9	21.8 ± 1.8	30.0 ± 5.0	<0.001***
Sex (female), n (%)	193 (39.2%)	98 (40.0%)	95 (38.5%)	0.727	31 (38.3)	85 (35.4)	77 (45.3)	0.120
**Score, mean ± SD**								
APACHE II	22.4 ± 7.7	27.3 ± 5.8	17.4 ± 6.1	<0.001***	22.9 ± 7.8	21.9 ± 7.3	22.7 ± 8.3	0.660
Charlson comorbidity index	4.9 ± 2.8	5.7 ± 2.6	4.2 ± 2.9	<0.001***	6.9 ± 3.3	8.0 ± 3.7	5.1 ± 2.8	0.849
SOFA	8.1 ± 3.9	10.2 ± 3.4	6.8 ± 3.5	<0.001***	8.2 ± 3.5	8.9 ± 3.7	8.9 ± 4.2	0.002**
mNUTRIC	5.3 ± 1.9	6.9 ± 0.9	3.8 ± 1.2	<0.001***	5.2 ± 1.9	5.2 ± 1.9	5.5 ± 1.9	0.204
Comorbidities (number)	2.0 ± 1.2	2.4 ± 1.1	1.6 ± 1.2	<0.001***	1.8 ± 1.1	1.9 ± 1.2	2.2 ± 1.2	0.017*
**Admission days, mean ± SD**								
ICU days	11.9 ± 10.4	13.5 ± 11.5	10.3 ± 8.8	<0.001***	11.0 ± 6.6	11.5 ± 11.6	12.7 ± 9.9	0.041*
LOS	35.8 ± 31.4	38.5 ± 33.3	33.0 ± 29.1	0.055†	35.8 ± 31.1	37.0 ± 33.1	34.0 ± 28.9	0.815
**Comorbidities, n (%)**								
Coronary artery disease	63 (12.8)	34 (13.9)	29 (11.7)	0.478	6 (7.4)	24 (10.0)	33 (19.4)	0.005**
History of stroke	52 (10.6)	34 (13.9)	18 (7.3)	0.017*	9 (11.1)	28 (11.6)	15 (8.8)	0.653
Hypertension	269 (54.7)	158 (64.5)	111 (44.9)	<0.001***	36 (44.4)	124 (51.5)	109 (64.1)	0.005**

COPD	76 (15.4)	40 (16.3)	36 (14.6)	0.591	22 (27.2)	32 (13.2)	22 (12.9)	0.006**
Cancer	141 (28.7)	74 (30.2)	67 (27.1)	0.450	25 (30.9)	69 (28.6)	47 (27.6)	0.870
CKD	141 (28.7)	100 (40.8)	41 (16.6)	<0.001***	19 (23.5)	68 (28.2)	54 (31.8)	0.145
Liver cirrhosis	39 (7.9)	23 (9.4)	16 (6.5)	0.232	5 (6.2)	18 (7.5)	16 (9.4)	0.630
Diabetes mellitus	199 (40.4)	126 (51.4)	73 (29.6)	<0.001***	30 (37.0)	89 (36.9)	80 (47.1)	0.095
**Mortality, n (%)**								
7-day mortality	25 (5.1)	17 (6.9)	8 (3.2)	0.062†	1 (1.2)	16 (6.6)	8 (4.7)	0.154
28-day mortality	101 (20.5)	60 (24.5)	41 (16.6)	0.030*	18 (22.2)	42 (17.4)	41 (24.1)	0.234
90-day mortality	174 (35.4)	112 (45.7)	62 (25.1)	<0.001***	26 (32.1)	78 (32.4)	70 (41.2)	0.147

***$p<0.001$; **$p<0.01$; *$p<0.05$; †$p<0.09$

Abbreviations: SD, standard deviation; mNUTRIC, modified Nutrition Risk in Critically ill; BMI, body mass index; APACHE, acute physiology and chronic health evaluation; SOFA, sequential organ failure assessment; COPD, chronic obstructive pulmonary disease; CKD, chronic kidney disease; HbA1c, haemoglobin A1c; ICU, intensive care unit; LOS, length of stay

Table S2. Baseline characteristics and outcomes of patients with sepsis who had low modified NUTRIC scores in the validation cohort of the study

	mNUTRIC score ≤5 (n=247)	Underweight (n=46)	Normal-weight (n=126)	Overweight (n=75)	*p*
**Demographic characteristics, mean ± SD**					
Age (years)	64.7 ± 15.6	68.8 ± 15.6	62.7 ± 16.1	65.4. ± 14.3	0.164
BMI, kg/m^2	23.3 ± 6.3	15.8 ± 1.8	21.8 ± 1.8	30.5 ± 5.7	<0.001***
Sex (female), n (%)	95 (38.5%)	17 (37.0)	42 (33.3)	36 (48.0)	0.115
**Score, mean ± SD**					
APACHE II	17.4 ± 6.1	18.7 ± 5.8	17.7 ± 6.4	16.2 ± 5.7	0.054†
Charlson comorbidity index	4.2 ± 2.9	4.5 ± 3.0	4.0 ± 2.7	4.3 ± 2.5	0.682
SOFA on day 1	6.8 ± 3.5	5.1 ± 2.1	6.2 ± 3.4	6.3 ± 3.3	0.153
SOFA on day 3	5.1 ± 3.3	4.8 ± 2.6	5.2 ± 3.4	5.3 ± 3.5	0.938
SOFA on day 7	3.6 ± 3.2	3.2 ± 2.3	3.5 ± 2.3	4.0 ± 3.5	0.577
mNUTRIC	3.8 ± 1.2	4.1 ± 1.1	4.1 ± 1.0	4.0 ± 1.1	0.942
Comorbidities (number)	1.6 ± 1.2	1.5 ± 1.1	1.4 ± 1.1	2.0 ± 1.2	0.006**
**Comorbidities, n (%)**					
Coronary artery disease	40 (14.0)	2 (4.3)	9 (7.1)	18 (24.0)	<0.001***
History of stroke	28 (9.8)	3 (6.5)	8 (6.3)	7 (9.3)	0.716
Hypertension	126 (44.4)	18 (39.1)	46 (36.5)	47 (62.7)	0.001**
COPD	34 (11.9)	11 (23.9)	15 (11.9)	10 (13.3)	0.133
Cancer	62 (21.8)	13 (28.3)	39 (31.0)	15 (20.0)	0.236
CKD	42 (14.7)	9 (19.6)	14 (11.1)	18 (24.0)	0.050†

Liver cirrhosis	19 (6.7)	3 (6.5)	8 (6.3)	5 (6.7)	0.996
Diabetes mellitus	85 (29.8)	15 (30.4)	30 (23.8)	29 (38.7)	0.082†
**Admission days, mean ± SD**					
ICU days	10.3 ± 8.8	9.4 ± 5.2	10.0 ± 9.3	11.2 ± 9.7	0.360
LOS	33.0 ± 29.1	31.7 ± 25.0	32.8 ± 30.2	34.2 ± 30.0	0.930
**Mortality, n (%)**					
7-day mortality	8 (3.2)	1 (2.2)	6 (4.8)	1 (1.3)	0.374
28-day mortality	41 (16.6)	8 (17.4)	21 (16.7)	12 (16.0)	0.980
90-day mortality	62 (25.1)	11 (23.9)	34 (27.0)	17 (22.8)	0.775

***p<0.001; **p<0.01; *p<0.05; †p<0.09

Abbreviations: SD, standard deviation; mNUTRIC, modified Nutrition Risk in Critically ill; BMI, body mass index; APACHE, acute physiology and chronic health evaluation; SOFA, sequential organ failure assessment; COPD, chronic obstructive pulmonary disease; CKD, chronic kidney disease; HbA1c, haemoglobin A1c; ICU, intensive care unit; LOS, length of stay
